# Symptomatic Effects of Exposure to Diluted Air Sampled from a Swine Confinement Atmosphere on Healthy Human Subjects

**DOI:** 10.1289/ehp.6814

**Published:** 2005-02-09

**Authors:** Susan S. Schiffman, Clare E. Studwell, Lawrence R. Landerman, Katherine Berman, John S. Sundy

**Affiliations:** ^1^Department of Psychiatry and; ^2^Department of Medicine, Duke University Medical Center, Durham, North Carolina, USA

**Keywords:** airborne emissions, attention, environmental chamber, memory, mood, nasal inflammation, pulmonary function, secretory immunity, spirometry, swine

## Abstract

Aerial emissions from a swine house at North Carolina State University’s field laboratory were diluted to a level that could occur at varying distances downwind from a confined animal feeding operation (CAFO) both within and beyond the property line, and these emissions were delivered to an environmental exposure chamber. The study design consisted of two 1-hr sessions, one in which 48 healthy human adult volunteers were exposed to diluted swine air and another in which they were exposed to clean air (control). Objective measures of blood pressure, temperature, heart rate, respiratory rate, lung function, nasal inflammation, secretory immunity, mood, attention, and memory were correlated with objective measures of air quality. Ratings of perceived (self-reported) health symptoms were also obtained. The mean levels of airborne constituents in the swine air condition were hydrogen sulfide (24 ppb), ammonia (817 ppb), total suspended particulates (0.0241 mg/m^3^), endotoxin (7.40 endotoxin units/m^3^), and odor (57 times above odor threshold). No statistical differences on objective measures of physical symptoms, mood, or attention resulted from the 1-hr exposure to swine emissions in the environmental chamber when compared with clean air for healthy human volunteers. However, subjects were 4.1 (*p* = 0.001) times more likely to report headaches, 6.1 (*p* = 0.004) times more likely to report eye irritation, and 7.8 (*p* = 0.014) times more likely to report nausea in the swine air (experimental) condition than in the control condition. These results indicate that short-term exposure in an environmental chamber to malodorous emissions from a swine house at levels expected downwind can induce clinically important symptoms in healthy human volunteers.

The rapid proliferation of confined animal feeding operations (CAFOs) that house thousands of animals at a single facility has raised public health concerns for workers as well as neighbors who live in adjacent communities (Schiffman et al. 1995; Thu et al. 1997; Wing and Wolf 2000). One focus of this concern has been potential human health effects from airborne agents that emanate from livestock houses, waste storage systems, and manure application sites. Aerial emissions from CAFOs are composed of a mixture of hydrogen sulfide (H_2_S), ammonia, volatile organic compounds (VOCs), and particulates including bioaerosols that arise during biodegradation of manure (Sweeten 1988). VOCs, ammonia, and H_2_S in the emissions are found in the gas phase as well as adsorbed to particulates (Schiffman 1998; Schiffman et al. 2001a).

Occupational studies of workers at CAFOs have documented a variety of health complaints as well as objective health effects including respiratory inflammation and dysfunction. Common health complaints among workers at animal production facilities include sinusitis, chronic bronchitis, nasal mucous membrane inflammation, nasal and throat irritation, headaches, and muscle aches and pains (University of Iowa Study Group 2002). Objective measures of lung function using spirometry have shown both acute cross-shift decline in lung function as well as chronic respiratory impairment in workers at confined swine and poultry feeding operations (Donham 1993; Donham et al. 1977; Schwartz et al. 1992, 1995). Progressive decline in lung function among CAFO workers occurs over a period of years. Furthermore, acute exposures to high levels of H_2_S from agitated manure can lead to reactive airway distress syndrome, permanent neurologic damage, and even death (Centers for Disease Control and Prevention 1993; Schiffman et al. 2001a).

Studies of potential health risks to community residents living in the proximity of CAFOs have been more limited than investigations of occupational risks. Several controlled studies in North Carolina and Iowa, however, have found that self-reported health symptoms are elevated in communities adjacent to intensive swine facilities. A field study in Iowa found that persons residing within a 2-mile radius of a 4,000-head swine operation reported higher frequencies of 14 out of 18 physical health symptoms, especially respiratory symptoms, than did a control group in an area with no intensive livestock operations (Thu et al. 1997). Residents of a rural North Carolina community with a 6,000-head hog operation reported increased symptoms of headache, runny nose, sore throat, excessive coughing, diarrhea, burning eyes, and reduced quality of life compared with residents in rural communities with intensive cattle operations or without livestock facilities (Wing and Wolf 2000). Furthermore, residents near swine facilities in North Carolina reported more tension, more depression, more anger, more fatigue, and more confusion at the time when the odors were strongest than did a control group of unexposed persons (Schiffman et al. 1995). No objective medical tests of physical health symptoms, however, were obtained in these community studies near CAFOs.

The purpose of the present investigation was to build upon previous occupational health and epidemiologic studies that have reported health symptoms associated with exposure to swine emissions. In this study, we used an exposure chamber to systematically investigate the physiologic and psychological responses in human volunteers that result from an exposure to a known level of emissions of swine confinement air in a controlled environment. The environmental chamber was built next to a swine facility, and air from a swine house was diluted to a level that could occur downwind from a confined swine operation both within and beyond the property line. This method of exposure was novel in that it enabled an assessment of the symptomatic effects of an environmentally relevant mixture of well-characterized pollutants in a group of self-selected healthy volunteers. The objective was to determine whether healthy human subjects voluntarily exposed to diluted air from a swine confinement house in a controlled environment (e.g., environmental chamber) experienced altered lung function, nasal inflammation, psychological changes, or other health symptoms related to such an exposure. Use of the human chamber allowed direct dose–response assessment of potential acute health effects from a specified level of airborne emissions.

## Materials and Methods

### Overview

The study design consisted of two 1-hr sessions, one in which human subjects were exposed to diluted swine air (experimental condition) and another in which they were exposed to cleaned air (control condition). Objective measures of lung function, nasal inflammation, secretory immunity, mood, respiratory symptoms, attention, and memory were correlated with objective measures of air quality. The concentration of odor, particulates, H_2_S, ammonia, VOCs, and endotoxin in exposure chamber air were monitored throughout the study. The maximum exposure duration of 1 hr was requested by the Duke Institutional Review Board because health complaints have been reported to North Carolina agencies from ≤ 1 hr of exposure.

### Subjects

Forty-eight healthy adults, ranging in age from 19 to 49 years (mean age = 26.0 ± 9.46 years), participated in this study. Half of the subjects were males and half were females. The group consisted of 33 Caucasians, 11 African Americans, 2 Hispanics, and 2 Asian Americans. The subjects were volunteers recruited by advertisements that were posted in workplaces throughout the Research Triangle community of North Carolina (Durham, Chapel Hill, and Raleigh). Potential subjects were prescreened by telephone to determine their eligibility for study participation. Those who met the inclusion criteria were enrolled sequentially in the order that they called. Enrollment stopped when 24 males and 24 females who met the inclusion criteria were enrolled.

To meet the criteria for the study, subjects were required to be healthy adults. Exclusion criteria were history of asthma (present or past), allergies for which they took prescription medications, smoking (not current smokers and never smoked > 10 packs of cigarettes in their lifetime), use of chronic prescription medications (except birth control pills), history of heart or lung disease, significant occupational exposure, and pregnancy. The mean height of the group was 67.4 ± 3.97 inches; the mean weight of the group was 171.4 ± 38.5 lbs. Subjects were paid $500.00 for their participation. All subjects signed a consent form approved by the Duke University Institutional Review Board that indicated their willingness to participate in “an experiment on exposure to air from swine operations.” All 48 subjects completed the study, and none experienced a serious adverse event.

### Exposure Facility

**Figure 1:**
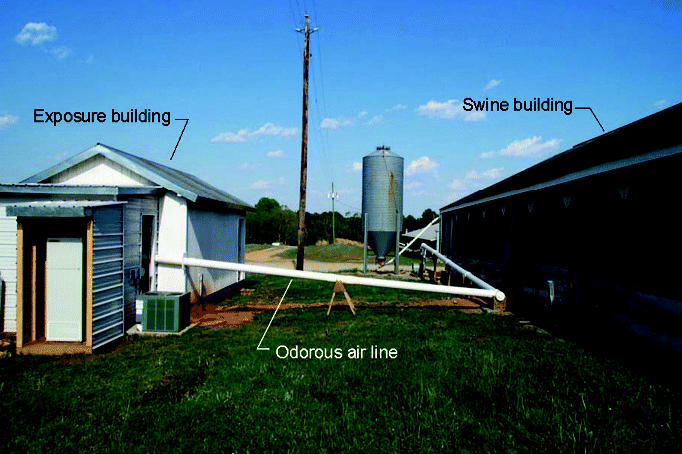
Exposure facility. Reprinted from Bottcher et al. 2002 with permission.

An exposure facility was constructed at the Swine Unit of North Carolina State University’s field laboratory. The exposure chamber (12 × 16 ft) was adjoined by a medical examination room 8 × 12 ft (Figure 1). The exposure chamber accommodated eight subjects who were seated at a table with dividers so that they could not speak to or observe each other (Bottcher et al. 2002). The ventilation system was custom designed so that it could deliver either totally cleaned air drawn from outside through an air cleaning unit (control condition) or emissions drawn from the exhaust fans of an adjacent swine building diluted with cleaned air (experimental condition). The cleaned air was generated from outside air processed by a packaged air-cleaning unit (model 6500; Allerair Industries, Laval, Québec, Canada) that consisted of an in-series arrangement of a prefilter and HEPA filter for particulate matter removal and two cartridges containing activated charcoal granules specially formulated for removal of gaseous pollutants expected from swine facilities. Particulates were not directly filtered or removed from the swine building air stream so that they would be incorporated into the exposure room airflow. The indoor airflow pattern within the exposure chamber was symmetrical in order to eliminate variability in air quality in the microenvironments of each of the eight subjects. The walls of the facility were insulated and paneled with waterproof materials that did not absorb odors and could be completely cleaned between trials to eliminate residual odorant compounds and particulates from surfaces. The air conditioning system was sufficient to maintain the chamber at a constant temperature (70°F) with eight subjects in the room.

### Exposure Conditions and Monitoring of Airborne Constituents

Subjects participated in two 1-hr exposures, one in which they were exposed to diluted swine air (experimental condition) and another in which they were exposed to cleaned air (control condition). The levels of gases, VOCs, particulates, endotoxin (a cell wall component of gram-negative bacteria), and odor in the experimental condition simulated concentrations that could occur downwind of swine production facilities both within and beyond the property line. Although higher concentrations than those tested here can potentially occur intermittently during sporadic spraying of fields with aerosolized liquid from the lagoons that hold decomposing waste, the levels used in this study are representative of air emissions both within and beyond the property line in the absence of spraying. Simulation of downwind exposure was achieved by the custom-designed air flow system that provided a variable method of mixing clean air with swine building air to allow a range of dilution ratios. The mean levels of the H_2_S, ammonia, particulates, endotoxin, and odor in the two exposure conditions are given in Table 1. All means in the experimental condition were significantly different from those in the control condition as determined by *t*-tests.

### Techniques to Quantify Airborne Emissions

H_2_S was measured continuously with a Jerome 631-X H_2_S analyzer (Arizona Instrument, Tempe, AZ) that uses a gold film sensor selective for H_2_S without interference from sulfur dioxide, carbon dioxide, carbon monoxide, and water vapors. Ammonia was measured continuously with the model 17C chemiluminescence ammonia analyzer (Thermo Environmental Instruments, Franklin, MS). Total suspended particulate concentrations were measured in real time by the HAZ-DUST EPAM-5000 environmental particulate air monitor (Environmental Devices Corporation, Haverhill, MA) that uses aerodynamic particle sizing and an in-line filter cassette for gravimetric sampling. Endotoxin was collected on fiberglass filters placed in a 47-mm in-line filter holder (model 2220; Gelman Sciences, Pall Corporation, East Hills, NY) connected to a piston pump that was calibrated at 46 L/min (Rietschle Thomas, Sheboygan, WI). The endotoxin was eluted from the filters with 15 mL deionized water. Endotoxin on the filters was quantified using a *Limulus* amebocyte lysate (LAL) assay (Bio-Whittaker, Walkersville, MD), and the concentration in endotoxin units (EU) was calculated (EU per milliliter). The concentration was multiplied by the elution volume to get the total EUs in the sample (total per filter). The concentration of endotoxin in the air was then calculated using the pump speed (46 L/min) and collection period (60 min). Odor levels in the chamber were measured in two ways. Real-time monitoring of odor levels was performed with the Scentometer (Barnebey-Sutcliffe, Columbus, OH) and the Nasal Ranger (St. Croix Sensory, Lake Elmo, MN). The Scentometer and Nasal Ranger are portable devices that can determine the number of dilutions necessary to reach threshold (i.e., odor dilution-to-threshhold; D/T). In addition, air samples from the exposure chamber were obtained in Tedlar bags during each trial, and odor thresholds were determined in the laboratory by a trained panel using an AC’SCENT olfactometer (St. Croix Sensory). The mean value for each of the above variables in a given condition was maintained within 8% of the overall mean in Table 1 for each test session. Variability within a session was also limited to 8% using a plenum in the inlet with data integrated over 5-min intervals.

VOCs were measured in two ways. First, real-time monitoring of VOCs at ppb levels was performed with the ppbRAE VOC monitor PGM-7240 (RAE Systems, Sunnyvale, CA) that uses a photoionization detector that can detect VOC concentrations down to a few parts per billion. Second, air samples were obtained in canisters and analyzed by gas chromatography and mass spectrometry (GC/MS) and gas chromatography/flame ionization detection (GC/FID) at the U.S. Environmental Protection Agency (EPA; Research Triangle Park, NC). Mean total VOCs were numerically elevated in the experimental condition compared with the control condition using both the ppbRAE and GC techniques, but this did not reach statistical significance. The mean exposure in the experimental condition as determined by GC/FID was 344.2 ± 27.6 ppbC (parts per billion carbon) and in the control condition, 322.7 ± 21.3 ppbC.

### Study Design

Each subject participated in two separate sessions that were at least 10 days apart. In one session, subjects were exposed to filtered air pumped into the exposure chamber for 1 hr (control session); in the other session, subjects were exposed for 1 hr to air from the swine house that had been diluted with uncontaminated air (experimental session). Eight subjects were tested at a time, resulting in 12 total sessions for all 48 subjects. Half the subjects participated in the experimental session first, and the other half participated in the control session first.

A series of physiologic and psychological measurements were obtained at four time points on each of the two exposure days: just before exposure, during the 1 hr exposure (at 30 min into the exposure), directly after exposure (at 1 hr), and 2 hr after the end of exposure (3 hr after beginning the exposure). The measurements assessed vital signs (blood pressure, temperature, heart rate, respiratory rate), pulmonary function (spirometry), nasal inflammation (using nasal lavage), total salivary IgA, mood [Profile of Mood States (POMS) scale (McNair et al. 1992)], attention, memory, and other symptoms. The timeline for these measurement types is given in Table 2.

#### Vital signs.

Blood pressure and heart rate were measured using a Dinamap Pro 100 monitor (GE Healthcare—Critikon Division, Jupiter, FL). A Welch Allyn SureTemp thermometer (model 679; Welch Allyn Medical Products, Skaneateles Falls, NY) with an oral probe and a disposable Welch-Allyn probe cover were used to measure temperature. Respiratory rate was determined by counting the number of breaths each subject took in a 30-sec time interval and then multiplying that number by 2.

#### Spirometry.

Forced vital capacity (FVC), forced expiratory volume at 1 sec (FEV_1_), and averaged forced expiratory flow between the full expiration of 25 and 75% of the total FVC (FEF 25–75%) were assessed in triplicate using a KoKo Portable Spirometer and KoKo Pulmonary Function Testing Software (PDS Instrumentation, Louisville, CO). FVC is the maximal volume of air (in liters) released during the forced maximal expiration. FEV_1_ is the volume of air that was expired in the first second of the forced maximal expiration. FEF 25–75%, measured in liters per second, gives an indication of the condition of the subject’s smaller airways. The pulmonary function testing software indicated which of the three trials was the best for each subject. The best trial from the preexposure testing was compared with the best trial from the postexposure testing to determine if there were any changes in the subjects’ pulmonary functioning. Subjects’ height and weight were measured and recorded at the first visit because this information was necessary to analyze the pulmonary function data.

#### Nasal lavage.

The nasal passages of study subjects were lavaged with 10 mL saline (0.9% sodium chloride; Abbott Laboratories, Chicago, IL), before and 3 hr after initiation of exposure (2 hr after completion of exposure) to swine facility air and to cleaned air. Subjects sat in a chair with their heads tilted back. They were given a plastic straw and instructed to blow into the straw while blocking the other end of the straw with a finger to close the passageway between the nose and the throat. Five milliliters of saline solution (warmed to body temperature) were introduced into each naris using a needleless 10-cc syringe and were held in the nares for 10 sec. The contents of the nares were then expelled into a 120-mL sterile specimen container. The sample was then transferred from the specimen container to a 15-mL centrifuge tube. The samples were put immediately on ice and transferred to the laboratory for analysis. Lavage fluids were treated with *N*-acetyl cysteine to disrupt mucus, and the cells were pelleted by centrifugation. Total cell counts were also determined by enumeration using a hemacytometer. Cytospin preparations of cells were stained for differential analysis. The nasal lavage supernatants were frozen at −70°C for cytokine analysis. The levels of the proinflammatory cytokines interleukin (IL)-1β and IL-8 were quantified because of their recognized importance in lipopolysaccharide-induced airway responsiveness (Jagielo et al. 1996; Wang et al. 1998). Both polymorphonuclear cells (PMN) and IL-8 are also known to increase dramatically in the lungs of persons who spend several hours inside of swine buildings (Larsson et al. 1997; Senthilselvan et al. 1997). Undiluted specimens of nasal lavage fluid were analyzed using Quantikine enzyme-linked immunosorbent assay (ELISA) kits (R&D Systems, Minneapolis, MN) for the proinflammatory cytokines IL-8 and IL-1β.

#### Salivary IgA.

Unstimulated saliva samples were collected using a sterile 2.0-mL vial and one-third of a plastic straw. Subjects uncapped the vial, placed the straw into the vial, and passively drooled down the straw for 90 sec. The samples were then collected and immediately placed in a freezer. They were later sent to Salimetrics LLC (State College, PA) on dry ice, where they were analyzed for salivary IgA. These measurements were obtained because Avery et al. (In press) found that persons exposed to strong swine odors had reduced levels of salivary IgA. All samples were assayed for salivary IgA in duplicate using a highly sensitive enzyme immunoassay (EIA) developed by Salimetrics. The test used 25 μL saliva, has a lower limit of sensitivity of 2.5 μg/mL, a range of sensitivity from 2.5 to 540 μg/mL, and average intra- and interassay coefficients of variation 5.6 and 8.79%, respectively. Method accuracy, determined by spike recovery, and linearity, determined by serial dilution, are 108 and 101%. Intermethod correlations for salivary IgA levels from saliva samples (*n* = 21) assayed using the Salimetrics EIA protocol and a radial diffusion assay, and the Salimetrics EIA protocol and a commercially available salivary IgA ELISA, were *r*(19)-values = 0.94 and 0.91 (*p*-values < 0.0001), respectively. The salivary IgA levels returned by the Salimetrics EIA protocol (mean ± SD = 379.39 ± 261.47 μg/mL) and the comparison ELISA (mean ± SD = 365.81 ± 311.53 μg/mL) were not statistically distinct. Salivary IgA levels returned by radial immunodiffusion were significantly higher (mean ± SD = 675.21 ± 467.94) than levels from both immunoassay protocols.

#### Mood.

The POMS questionnaire was used to assess mood. The POMS is a highly sensitive standardized scale that, based on subjects’ responses, measures six different aspects of transient mood: anger–hostility, confusion–bewilderment, depression–dejection, fatigue–inertia, tension–anxiety, and vigor–activity. The POMS has been used previously to evaluate mood changes in response to odors by neighbors of swine operations (Schiffman et al. 1995). The POMS questionnaire has been extensively tested and validated; it has been widely used to evaluate the degree to which behavioral and treatment interventions as well as environmental factors affect mood. The form of the scale used here consists of 30 different feelings (Appendix 1) on which subjects rated “how they are feeling at the present time” on scales coded from 0 (not at all) to 4 (extremely).

#### Attention and memory.

We used a digit span test to measure levels of attention and memory. The test was a modified version of the digit span test used on the Weschler Adult Intelligence Scale, in which a researcher reads strings of simple numbers to a subject, and the subject repeats the numbers back to the researcher in the correct order. The test was presented visually in the present study rather than orally so that the results were not affected by the qualitatively different voices of several researchers who administered the test. Each subject was presented with strings of simple numbers (from 1 to 9) using flashcards, beginning with a string of two digits and presenting one digit per second. After each string of numbers, the subject was shown a flashcard that read, “recall numbers.” The subject then recalled the digits in the order in which they were presented by writing them down. Each subject was given 10 sec between the time that they saw the “recall numbers” flashcard and the time that they were presented the next string of numbers to recall and write down the string of digits. After each recall, a new string of digits was presented, with each successive string increased by one digit until the subject recalled the last string consisting of 9 digits. Because the digit span test was administered to the subjects four times at each visit, four different sets of cards were made using random number generation. The same four sets were used at the subjects’ second visit, but the sets were presented to the subjects at different time points at the second visit. The subject’s score was the length of the last string of numbers accurately recalled.

#### Odor ratings.

The perceived odor was rated on three global 9-point line scales numbered from 0 to 8. These included odor intensity, irritation intensity, and hedonic ratings. For odor and irritation intensity, the scale was labeled as follows: 0, none at all; 1, very weak; 2, weak; 3, moderate weak; 4, moderate; 5, moderate strong; 6, strong; 7, very strong; and 8, maximal. The descriptors for pleasantness/unpleasantness were 0, extremely pleasant; 1, very pleasant; 2, moderately pleasant; 3, slightly pleasant; 4, neither pleasant nor unpleasant; 5, slightly unpleasant; 6, moderately unpleasant; 7, very unpleasant; and 8, extremely unpleasant. Subjects also rated an additional five scales to characterize the odor using the intensity scale above: “musty, earthy, moldy,” “fecal,” “like urine,” “sewer odor,” and “sweaty.”

#### Environmental Exposures and Health Questionnaire.

Subjects indicated how much, if at all, they were affected by 48 different symptoms on this questionnaire (Appendix 2). The Environmental Exposures and Health Questionnaire (EEHQ) was developed by the U.S. EPA Health Effects Research Laboratory and has been used previously to assess health symptoms from odors (Schiffman 1998). Subjects made their ratings on four different categories: don’t have symptom at all (0), mildly affected (1), moderately affected (2), severely affected (3).

#### Description of statistical methods.

For all but one outcome, we estimated two equations of the general form:


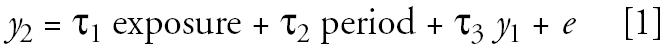



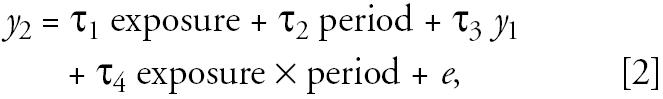


where *y*_1_ and *y*_2_ are the pre- and postexposure scores on an outcome, “exposure” is a dummy variable coded 1 for swine air and 0 otherwise, and “period” is a dummy variable coded 0 for those who received clean air first, and 1 for those who received swine air first. In Equation 1, the coefficient for exposure (τ_1_) estimates its effect on *y*_2_ with preexposure score and period-related differences controlled. As shown by Kessler and Greenberg (1981), this coefficient is equivalent to the effect of exposure on (time 2 – time 1) change in the dependent variable controlling for other independent variables in the equation. Our significance tests for the effect of exposure on each dependent variable are based on this coefficient from Equation 1. The (exposure × period) product term in Equation 2 was used to test whether the effect of exposure differed according to whether swine air was administered first or second. On all but one dependent variable (discussed below), this testfor the presence of a carryover effect was negative.

The analysis focused on potential effects of exposure on seven general classes of outcome variables: vital signs, pulmonary function (spirometry), nasal inflammation (cytokines and cell counts), saliva measures (salivary IgA), mood (POMS), memory/attention (digit span), and self-reported symptoms. Several of these classes, including vital signs, self-reported symptoms, mood, and digit span, contained multiple measures after exposure commenced. Because we did not hypothesize delayed effects of exposure on these specific outcomes, we tested whether exposure-related differences were present at multiple time points after exposure only if a significant effect was present for the first measurement after exposure. Given the exploratory nature of the study, we did not correct for multiple tests. However, given the *p*-values and magnitudes of most significant effects, the positive findings are not the result of chance. We return to this issue in the discussion of the findings.

All outcomes other than respiratory symptoms were analyzed as continuous dependent variables. We used SAS PROC MIXED (SAS Institute, Cary, NC) to obtain generalized least squares estimates of the coefficients (τ) in Equations 1 and 2, with between-subject variance treated as a random effect and removed from the error term in significance testing. As discussed by Verbeke and Molenberghs (1997), generalized least squares estimators are more “efficient” (have smaller variance) compared with corresponding ordinary least squares estimators.

On self-reported symptom measures, nearly all respondents had scores of 0 or 1. Therefore, each self-reported symptom measure was coded as a (0/1) variable scored 1 for the presence of any symptoms, and SAS PROC GENMOD was used to estimate Equations 1 and 2 as logistic generalized estimating equations. Between-subject variance was again treated as a random effect, making these models the logistic equivalent of those estimated in PROC MIXED for the continuous outcomes. To examine potential non-proportionality (nonequivalence) of effects between those with and those without self-reported symptoms at baseline, we performed two analyses for self-reported symptoms.

## Results

### Results of significance testing for effects of exposure on change in an outcome.

First, Equations 1 and 2 were estimated for all respondents. Then respondents reporting any preexposure symptoms were dropped, and our models were re-estimated excluding preexposure score (*y*_1_) as a control. [An average of four respondents was excluded across self-reported symptom outcomes (maximum = 12) in the second set of analyses]. Results were essentially the same for both logistic analyses. In Table 3 we report those based on the full sample of respondents. For each dependent variable, we present *p*-values for whether change in an outcome is significantly different in the exposure group compared with the control group. When significant differences are present, we give regression coefficients estimating the effect of exposure (vs. control) on change in a dependent variable. Unlike the raw group differences in the descriptive tables, these coefficients are estimated controlling for initial (preexposure) status and for period of exposure.

None of the measures of vital signs, pulmonary function (spirometry), nasal lavage, salivary IgA, mood, or digit span score was significantly related to exposure. Two nasal lavage measures were related to exposure. Compared with controls, the (time 1–time 2) decrease in percentage of epithelial cells was greater among those exposed to swine air. The exposure group also had a larger increase in percentage of lymphocytes but not in absolute numbers of lymphocytes. Three (of 11) measures of the self-reported symptoms were significantly related to exposure. Based on the logistic odds ratio, when subjects were exposed to swine air, they were 4.1 (*p* = 0.001) times more likely to report headaches, 6.1 (*p* = 0.004) times more likely to report eye irritation, and 7.8 (*p* = 0.014) times more likely to report nausea than in the control condition. Significant exposure-related differences on headache were still present at time 3. None of the pulmonary or mood measures was related to exposure.

### Descriptive statistics.

The means ± SDs for physical measures (vital signs, nasal lavage, salivary measures, and pulmonary function) over time are given in Table 4. Results of pulmonary function studies are presented as percentage of predicted values based upon population norms. It is customary to report the magnitude of change as percent change from baseline. Means ± SDs for scores on POMS at four time points are shown in Table 5. Means ± SDs for scores on digit span at four time points are shown in Table 6. Table 7 gives the number of persons who self-reported specific symptoms.

### Odor perception.

All subjects perceived an odor in the experimental condition and very little odor in the control condition, with no overlap of ratings in the two conditions by any subject. The mean odor intensity during the experimental exposure was 5.29 (moderate strong to strong) compared with 1.46 (very weak to weak) in the control condition. The mean irritation intensity during the experimental exposure was 3.77 (moderate weak to moderate) compared with 0.73 (very weak) in the control condition. The mean unpleasantness during the experimental exposure was 6.21 (moderately unpleasant to very unpleasant) compared with 4.12 (neither pleasant nor unpleasant to slightly unpleasant) in the control condition. The rank order of the mean intensities on the odor adjectives in the experimental condition was fecal > sewer odor > musty, earthy, moldy > like urine > sweaty.

## Discussion

The results of this study indicate that a 1-hr exposure to odorous swine air in an environmental chamber (at levels that could occur downwind from a swine facility both within and beyond the property line) has no significant acute effects on vital signs, lung function, nasal inflammation, salivary IgA, mood, attention, or memory in healthy volunteers. That is, there were no statistical differences on objective measures of physical symptoms, mood, or attention that resulted from a 1-hr exposure to air emissions from a swine facility when compared with clean air in persons who self-selected to participate in the exposure study. However, self-reported symptoms of headaches, eye irritation, and nausea were significantly more prevalent in these healthy volunteers exposed to swine air for 1 hr compared with clean air. The rapid onset of exposure-related avoidance symptoms reported by our subjects in response to diluted swine air is consistent with epidemiologic studies (Thu et al. 1997; Wing and Wolf 2000) in which persons “downwind” from swine facilities report similar symptoms.

The underlying mechanism responsible for the headaches, eye irritation, and nausea is not known, but it is unlikely that a single constituent of the emissions induces these effects. As explained below, no single component in the airborne emissions was present at a high enough concentration to be wholly responsible for these symptoms. However, additivity or synergy among the combined components may be the cause of these physical symptoms (Schiffman et al. 2000). That is, the symptoms may be caused by the combined load of some or all of the components in the air (H_2_S, ammonia, VOCs, particulates, and endotoxin). Another possibility is that these self-reported symptoms are innate or learned warning signals of potential health effects at higher concentrations or with prolonged exposure.

### Endotoxin.

Headache, eye irritation, and nausea have been reported in previous studies by persons exposed to endotoxin (Crook et al. 1991; Melbostad and Eduard 2001; Poulsen et al. 1995a, 1995b; Thorn and Kerekes 2001). Endotoxin is also known to contribute to airway inflammation and airflow obstruction (Kline et al. 1999). However, it is unlikely that the endotoxin levels experienced by the subjects in this study are wholly responsible for these self-reported symptoms. The levels of endotoxin to which the subjects were exposed in the chamber were orders of magnitude lower than levels inside swine buildings (e.g., 3,984 EU/m^3^ reported by Zhang et al. 1998). Furthermore, the levels used in the experimental condition are far lower than ambient air endotoxin in office buildings (0.25–0.4 μg/m^3^) that have been associated with health complaints (Teeuw et al. 1994). (If one assumes that the biologic activity per mass unit of endotoxin is 10 EU/ng in this study, the exposure is approximately 0.06 ng/m^3^ in the clean air condition and 0.74 ng/m^3^ in the experimental condition.)

The cumulative exposure to endotoxin over 1 hr in the experimental condition of this study is also far below the level expected to cause physiologic symptoms. Assuming a tidal volume of 0.5 L (a single breath in normal quiet breathing) and 15 breaths/min, this translates to 450 L in 1 hr. Because there are 1,000 L in 1 m^3^, the cumulative dose in this study is 0.332 ng. This dosage is far below the 15–20 μg dose at which airway responsiveness is altered in sensitive populations (Michel et al. 1989) and the 40 μg dose at which airway resistance is altered in healthy, nonatopic, nonasthmatic controls (Kline et al. 1999).

### Ammonia.

The mean concentration of ammonia in the experimental arm of this study was 817 ppb, a concentration that is below the published eye irritation threshold (irritation just barely noticeable) for ammonia of 4 ppm (World Health Organization 1986). It is also far below the short-term (15 min) exposure limit of for ammonia of 35 ppm set by the Occupational Safety and Health Administration (OSHA 2003). Average concentrations of ammonia in swine housing have been reported to range from 5 to 18 ppm; maximum concentrations in sow buildings are 43.7 ppm and in finishing barns are 59.8 ppm (Koerkamp et al. 1998), but these levels decrease rapidly downwind as they are diluted in ambient air.

### H_2_S and VOCs.

H_2_S is a colorless, flammable gas that smells like “rotten eggs” at low concentrations. The mean concentration of H_2_S during the 1-hr exposure in this study was 24 ppb. This level is above the odor detection threshold (0.5 ppt to 8 ppb) but far below the irritant threshold, which ranges from 2.5 to 20 ppm (Amoore 1985; Collins and Lewis 2000). Thus, the H_2_S level in this study was 3–4 orders of magnitude (i.e., 10^3^ and 10^4^ times) below the level that causes classical irritant symptoms. The scientific literature on H_2_S, however, suggests that health symptoms can occur at H_2_S concentrations far below the levels at which irritation or toxicity occur. For example, community investigations near paper mills, refineries, geothermal sources, and meat-packing plants indicate that sustained exposure to low levels of H_2_S or other reduced sulfur compounds (below the irritant threshold) can cause health symptoms (Campagna et al. 2000; Jaakkola et al. 1990, 1991; Kilburn and Warshaw 1995; Legator et al. 2001). In two of these community studies, health symptoms were found from an average daily exposure to 10–11 ppb H_2_S (Jaakkola et al. 1990; Kilburn and Warshaw 1995).

GC/MS was performed on air samples from both the experimental and control conditions in our study, and many diverse compounds were identified in both the control and experimental conditions. The vast majority of these compounds were present at concentrations far below published odor thresholds; furthermore, all of the compounds for which irritation thresholds were available in the literature were below these levels (Schiffman et al. 2001b). Yet human assessments indicated that odors as well as irritant sensations were perceived in the exposure condition of this study. Comparison of the findings from chemical and human assessments in this study with previous studies (Cometto-Muñiz et al. 1997; Schiffman et al. 2001b) points to the importance of the cumulative effects of hundreds of compounds in producing odor and irritant sensations.

### Self-reported headaches, eye irritation, and nausea.

The underlying cause of the significant increase in self-reported headaches, eye irritation, and nausea in the experimental condition of this study is not known. As described above, no single component in the airborne emissions was present at a high enough concentration to be wholly responsible for these symptoms. It is possible, however, that synergy among the combined components may induce these physical symptoms. That is, the symptoms may be caused by the combined load of all or some of the components in the air (H_2_S, ammonia, VOCs, particulates, and endotoxin). Donham and Cumro (1999) have previously found that ammonia and particulates are synergistic with one another in their impact on human health. Furthermore, low concentrations (even sub-threshold levels) of individual VOCs can add together when delivered in a mixture to produce noticeable sensory irritation (Cometto-Muñiz et al. 1997, 1999; Korpi et al. 1999). Another possibility is that these self-reported symptoms are innate or learned warning signals of potential health effects at higher concentrations or with more prolonged exposure. The symptoms may carry more significance for health effects in studies of vulnerable populations, such as children and elderly, and patients with cardiovascular or respiratory diseases.

### Vital signs.

The finding that no significant changes in respiratory rate, blood pressure, or pulmonary function were found here suggests that a single 1-hr exposure to unpleasant swine odor typical of downwind concentrations does not impair these health parameters in healthy volunteers tested in an environmental chamber. Previous studies have shown that exposure to unpleasant odors can in some cases lead to an inhibited breathing pattern (Schiffman et al. 2000). Stress, independent of unpleasant odors, also produces sustained inhibited breathing patterns that in turn can elevate blood pressure (Anderson 1998; Anderson and Chesney 2002). The mediating mechanism for elevated blood pressure from sustained inhibition of respiration is acidification of the plasma with subsequent increases in sodium/hydrogen exchange in kidneys and blood vessels. If inhibited breathing did occur during the 1-hr exposure in this study, it was not sustained after exposure, nor was the breathing frequency sufficiently altered to affect blood pressure. Future studies may employ additional measures of cardiovascular function such as alteration in heart rate variability, a finding that is associated with adverse effects in relationship to air pollution. More sensitive markers of airway inflammation, such as increased exhaled nitric oxide or increased epithelial permeability, may yield clues to long-term health effects of swine air exposure.

### Mood (POMS scales).

The finding that a 1-hr exposure to odorous swine air had no significant effects on mood scores on the POMS scale of healthy volunteers tested in an environmental chamber contrasts with a previous community study in which neighbors were frequently exposed to swine odor (Schiffman et al. 1995). In that study, neighbors of swine facilities in North Carolina experienced significantly more tension, depression, anger, fatigue, and confusion and less vigor on POMS scales when odors were present than when odors were absent (Schiffman et al. 1995). The difference in these findings can be explained by the differences in the exposure situations and the persons exposed. In the present study using a chamber, subjects were healthy volunteers who knew that the exposure would be time-limited and that the exposure levels were controlled by the investigators and approved for human subjects by the Duke University Medical Center Institutional Review Board. Furthermore, they were financially compensated and could withdraw at any time. Neighbors of swine operations, however, have no advanced warning about the timing, magnitude, or duration of the exposure. The intermittent presence of unavoidable, and unpredictable malodors can engender feelings of lack of control and negative affect when neighbors cannot use their home and property as they want. Unpleasant odors in the home can affect overall quality of life. Unconscious odor conditioning may also play a role in impaired mood of neighbors. When odors are associated with stressful or unpleasant situations, this odor can elicit subsequently alter mood, attitudes, and behavior (Kirk-Smith et al. 1983).

### Salivary IgA.

The finding of no changes in salivary IgA concentrations in this study is probably due to the short duration of the exposure period as well as the fact that the subjects were healthy volunteers who were financially compensated. Participants in this experimental trial as volunteers had more control over the odor exposure than do persons actually living downwind of a swine facility. Previous studies have shown that unavoidable stress and passive coping can produce decrements in salivary IgA within 10–15 min, whereas active coping and controllable stressors can increase salivary IgA (Bosch et al. 2001; Ring et al. 2002; Willemsen et al. 2002). Real-life stressful events and negative emotions can also decrease salivary IgA (Carins and Booth 2002; Yang et al. 2002). A recent study in North Carolina of neighbors of swine facilities found that their salivary IgA decreases significantly upon exposure to moderately strong swine odors (Avery et al., in press). This indicates that unavoidable and unpredictable odors from swine facilities that are not time-limited can have psychophysical impacts. The long-term health significance of alterations in salivary IgA levels is not well understood at present.

### Odor ratings.

The mean intensity ratings of 5.29 for odor (moderate strong to strong) and 3.77 for irritation (moderately weak to moderate) given by naive subjects in the experimental condition (for an odor 56 times above threshold) are higher than those given for the same level of swine odor by trained panelists who have extensive experience rating swine odor both on and off of farms in a natural environment. Trained panelists rate an odor 56 times above threshold at a mean odor intensity of 4.21 (moderate to moderately strong) with an irritation intensity of 3.01 (moderately weak) (Schiffman and Graham 2004). The mean unpleasantness ratings given by naive subjects during the experimental condition to an odor of 56 odor units was 6.21 (moderately unpleasant to very unpleasant). Trained panels give this odor a mean rating of 5.76 (moderately unpleasant). The probable reason why trained panelists give lower numbers is context specific. Trained panelists are exposed to very intense odors at odor sources next to the barns and lagoons as well as odors downwind. That is, scores of trained panelists are based on a wider range of intensities.

## Conclusion

In this study that evaluated healthy volunteers, no statistical differences on objective physical measures, mood, or attention were found from a 1-hr exposure in an environmental chamber to air emissions from a swine house when compared with clean air. However, self-reported symptoms of headaches, eye irritation, and nausea were significantly higher in the swine air (experimental) condition than the clean air (control) condition. The underlying cause of self-reported headaches, nausea, and eye irritation in the experimental condition is not known but may be due to the combined load of some or all of the components in the air (H_2_S, ammonia, VOCs, particulates, and endotoxin). Another possibility is that these self-reported symptoms are innate or learned warning signals of potential health effects at higher concentrations or with prolonged exposure.

The self-reported headaches, nausea, and eye irritation in this controlled study using healthy volunteer subjects without occupational exposure are a subset of a larger number of symptoms reported in community studies by individuals exposed to environmental odors (Shusterman 1992; Thu et al. 1997; Wing and Wolf 2000). The greater number of health symptoms reported by neighbors of swine operations compared with our healthy volunteers may be due to inclusion of vulnerable populations (e.g., persons with asthma), previous exposure history, higher levels of exposure in certain communities (both swine and non-swine sources), involuntary and prolonged exposure, and quality of life issues. In addition, persons living downwind are exposed to emissions from lagoons and spray fields as well as swine houses, although the former two sources tend to contain similar but less varied compounds than those emitted from the houses (Schiffman et al. 2000).

More research is necessary to determine the mechanism responsible for self-reported symptoms and their elevated number in neighbor exposures relative to this experimental exposure. First, controlled studies in the environmental chamber should be expanded in the future to include volunteers from vulnerable populations (e.g., persons with asthma). Most scientific literature (Nieto et al. 2003; Nolte and Berger 1983; Sant’Ambrogio 1987; Shusterman 2002), but not all (Levi et al. 1990; Opiekun et al. 2003), suggests that persons with asthma have sensory hyper-responsiveness to irritants. These conflicting findings may be due to medical status at the time of testing; activation of afferent neurons in the airways is not a static property but rather appears to change rapidly in response to inflammation (Carr and Undem 2001). Asthmatic subjects with active symptoms may not volunteer for an exposure experiment.

Second, the contribution of stress must be incorporated in controlled experimental paradigms because stress responses can sensitize various neuronal, hormonal, and behavioral responses that could potentially affect the parameters tested in the present controlled exposure study (Johnson et al. 2004). Neighbors who are involuntarily exposed to unpredictable swine emissions report significantly more tension, depression, anger, fatigue, and confusion and less vigor on POMS scales (Schiffman et al. 1995) than did the subjects in the present experiment, whose exposure was voluntary. Although it is not possible to design a study that precisely replicates the involuntary and unpredictable exposure to malodorous swine emissions (potential stressor) in a natural setting, symptoms can be studied during a prolonged intermittent (and thus unpredictable) but time-limited exposure under controlled experimental paradigms. In addition, symptoms during exposure to swine air while performing a stressful activity (e.g., mental arithmetic) versus symptoms while performing a nonstressful activity (control) can be compared.

Controlled exposure studies as well as further epidemiologic studies should include subjects with a broad range of exposure history to swine emissions to determine the prevalence of sensitization as well as tolerance for (or adaptation to) odorous emissions. Several experimental studies suggest that increased sensitivity to an odor can develop with repeated exposure (Wysocki et al. 1989), and that the effect is pronounced in women (Dalton et al. 2002). Yet tolerance to swine confinement air (with fewer symptoms) has been reported to occur in some chronically exposed workers (Von Essen and Romberger 2003), although it is not known whether tolerance to aerial emissions develops in an analogous manner at lower concentrations that occur at neighbors downwind of swine facilities. Both controlled and epidemiologic research studies will help clarify the impact of sporadic exposure to swine emissions on health symptoms of persons who are involuntarily exposed intermittently to malodors.

## Figures and Tables

**Table 1:** Mean levels of the H_2_S, ammonia, particulates, endotoxin, and odor in the two conditions along with the instruments used for air quality measurements.

Emission	Instrument used for measurement	Control condition	Experimental condition
H_2_S	Jerome meter	0 ppb	24 ppb
Ammonia	Chemiluminescence analyzer	46.4 ppb	817 ppb
Total suspended particulates	HAZ-DUST	0.0136 mg/m^3^	0.0241 mg/m^3^
Endotoxin	LAL assay	0.63 EU/m^3^	7.40 EU/m^3^
Odor	Scentometer and nasal ranger	0.3 D/T*^a^*	56 D/T
Odor	AC’SCENT olfactometer	—	57 D/T

a D/T (dilutions to threshold) indicates the dilution ratio at which the sample has a probability of 0.5 of being detected under the conditions of the test.

**Table 2:** Timelines for physiologic and psychological measurements.

Just before exposure	30 min into exposure	1 hr (end of exposure)	2 hr after end of exposure
Vital signs*^a^*		Vital signs	Vital signs
Spirometry		Spirometry	
Nasal lavage			Nasal lavage
Salivary IgA		Salivary IgA	
Mood	Mood	Mood	Mood
Attention and memory	Attention and memory	Attention and memory	Attention and memory
Odor ratings	Odor ratings	Odor ratings	
EEHQ		EEHQ	EEHQ

EEHQ, Environmental Exposures and Health Questionnaire.

a Blood pressure, temperature, heart rate, respiratory rate.

**Appendix 1:** The 30 feelings that were rated on the POMS.

Tense	Unworthy	Gloomy
Angry	Uneasy	Sluggish
Worn out	Fatigued	Weary
Lively	Annoyed	Bewildered
Confused	Discouraged	Furious
Shaky	Nervous	Efficient
Sad	Lonely	Full of pep
Active	Muddled	Bad tempered
Grouchy	Exhausted	Forgetful
Energetic	Anxious	Vigorous

**Appendix 2:** The 48 symptoms on the EEHQ.

Hives, itching skin	Feeling angry, irritable	Feeling anxious, panicky
Skin rash	Feeling depressed	Wheeze, chest tightness
Skin redness, flushing	Eyes dry, irritated	Shortness of breath
Feeling feverish, chills	Tearing eyes	Chest pain
Migraine headache	Blurred vision	Heart racing, pounding
Sinus headache	Sinus/nasal congestion	Difficulty breathing
Other headache	Nasal secretions	Cough
“Spacy” feeling	Nasal irritation, burning	Cough up sputum, phlegm
Brain fog	Difficulty concentrating	Hoarseness
Cold hands or feet	Memory problems	Nausea
Throat sore, irritated	Inappropriate emotions	Vomiting
Throat itching inside	Ear redness, flushing	Diarrhea
Coordination problems	Ears itching inside	Abdominal bloating, pain
Muscle weakness	Daytime sleepiness	Constipation
Muscle aches, joint pain	Undue fatigue	Heartburn
Numbness of legs, arms	Trembling, body shaking	Pelvic pain

**Table 3:** Results of significance testing for effects of exposure on change in an outcome (effect coefficients are given for significant effects only).

	Group differences on time 2 – time 1 change		
	*p*-Value	Coefficient	*p*-Value, group differences at time 3*^a^*
Vital signs			
Heart rate	0.78		
Respiratory rate	0.57		
Temperature	0.27		
Systolic blood pressure	0.70		
Diastolic blood pressure	0.29		
Blood pressure ratio (systolic to diastolic)	0.52		
Spirometry			
Percent change FEV_1_	0.98		
Percent change FVC	0.80		
Percent change FEF 25–75%	0.88		
Salivary measure			
Salivary IgA (μg/mL)	0.57		
Mood scores (POMS)			
Anger	0.97		
Confusion	0.83		
Depression	0.45		
Fatigue	0.52		
Anxiety	0.39		
Vigor	0.52		
Total mood	0.55		
Digit span test			
Digit span score	0.35		
Nasal lavage			
IL-8 (pg/mL)	0.11		
IL-1β(pg/mL)	0.38		
Cell counts	0.76		
Percent epithelial cells	0.02	(b = −21.1)*^b^*	—*^c^*
Percent lymphocytic cells	0.008	(b = 23.0)	—*^c^*
Percent PMNs	0.22		
Absolute epithelial cells	0.15		
Absolute lymphocytic cells	0.78		
Absolute PMNs	0.27		
Self-reported symptoms			
Headache	0.001	(OR = 4.1)*^d^*	0.01
Sore throat	0.27		
Itchy throat	0.12		
Eyes irritated	0.004	(OR = 6.1)	0.07
Eyes tearing	—*^e^*		
Nasal congestion	0.76		
Nasal secretion	0.22		
Nasal irritation	0.34		
Difficulty breathing	—*^e^*		
Cough	0.66		
Nausea	0.014	(OR = 7.8)	0.57

a The *p*-value for time 3 is based on a test of whether the (time 2 – time 1) group differences persist at time 3. The time 3 test was performed only when group differences on (time 2 – time 1) were statistically significant.

b The b-coefficient obtained from SAS PROC MIXED represents the metric effect of exposure on an outcome at time 2 controlling for period and preexposure (time 1) differences.

c No time 3 measures were obtained for these outcomes.

d The odds ratio (OR) coefficient estimated with SAS PROC GENMOD is the exponentiated logistic effect of exposure on the odds of any symptom at time 2 controlling for period and preexposure differences.

e Model did not converge because of low prevalence at time 2.

**Table 4:** Means ± SDs for vital signs, salivary measures, nasal lavage, pulmonary function, and the digit span test over time.

	Condition	Before exposure	1 hr (end of exposure)	2 hr after end of exposure
Vital signs				
Heart rate	Experimental	70.85 ± 14.61	65.02 ± 13.36	65.79 ± 11.95
	Control	69.96 ± 11.49	64.73 ± 13.39	64.81 ± 12.07
Respiratory rate	Experimental	17.50 ± 4.24	17.25 ± 3.86	16.63 ± 4.84
	Control	17.04 ± 3.67	16.75 ± 3.19	16.88 ± 3.25
Temperature	Experimental	97.97 ± 0.74	97.85 ± 0.64	97.63 ± 0.50
	Control	97.83 ± 0.61	97.72 ± 0.66	97.57 ± 0.58
Systolic blood pressure	Experimental	122.27 ± 15.27	120.44 ± 15.67	123.88 ± 14.61
	Control	121.63 ± 15.32	119.73 ± 14.72	121.85 ± 15.35
Diastolic blood pressure	Experimental	66.44 ± 10.23	66.33 ± 10.02	67.52 ± 11.42
	Control	64.15 ± 10.71	65.33 ± 10.60	69.13 ± 9.62
Nasal lavage				
IL-8 (pg/mL)	Experimental	396.1 ± 448.4	NA	190.6 ± 213.0
	Control	385.0 ± 321.7	NA	268.4 ± 310.2
IL-1β(ng/mL)	Experimental	10.6 ± 21.8	NA	3.5 ± 8.3
	Control	4.6 ± 6.5	NA	4.6 ± 10.3
Cell counts	Experimental	205541.7 ± 442500.2	NA	240364.6 ± 505983.6
	Control	146937.5 ± 332148.5	NA	277354.2 ± 1155336.9
Percent epithelial cells	Experimental	55.6 ± 38.1	NA	35.7 ± 35.6
	Control	67.2 ± 40.1	NA	56.7 ± 40.6
Percent lymphocytic cells	Experimental	44.1 ± 38.0	NA	64.9 ± 35.6
	Control	32.6 ± 40.2	NA	42.0 ± 41.5
Percent PMNs	Experimental	0.1 ± 0.5	NA	0.0 ± 0.3
	Control	0.0 ± 0.0	NA	1.2 ± 6.0
Salivary measures				
Salivary IgA (μg/mL)	Experimental	193.42 ± 112.17	191.94 ± 116.57	NA
	Control	194.68 ± 120.39	179.89 ± 116.88	NA
Pulmonary function				
Percent change FEV_1_	Experimental	NA	0.02 ± 0.04	NA
	Control	NA	0.00 ± 3.31	NA
Percent change FVC	Experimental	NA	0.05 ± 0.03	NA
	Control	NA	−0.13 ± 3.78	NA
Percent change FEF 25–75%	Experimental	NA	1.02 ± 0.12	NA
	Control	NA	0.78 ± 7.63	NA

NA, not applicable.

**Table 5:** Means ± SDs for scores on POMS at four time points.

Group	Mood scale	Just before exposure	30 min into exposure	1 hr (end of exposure)	2 hr after end of exposure
Experimental	Anger–hostility	0.96 ± 1.86	1.42 ± 2.86	1.35 ± 3.27	0.94 ± 2.15
	Confusion–bewilderment	3.19 ± 1.83	3.79 ± 2.26	4.19 ± 2.47	3.60 ± 1.87
	Depression–dejection	0.83 ± 1.72	1.10 ± 2.43	1.02 ± 2.34	0.69 ± 1.69
	Fatigue–inertia	3.21 ± 3.96	4.79 ± 3.83	5.13 ± 4.29	4.15 ± 4.44
	Tension–anxiety	1.94 ± 2.93	1.73 ± 2.52	1.29 ± 2.20	0.79 ± 1.58
	Vigor–activity	8.27 ± 4.74	3.60 ± 3.75	3.29 ± 3.35	3.79 ± 3.87
	Total mood score	1.85 ± 11.71	9.23 ± 12.55	9.69 ± 12.56	6.38 ±10.80
Control	Anger–hostility	0.50 ± 1.46	1.00 ± 2.79	0.94 ± 2.68	1.17 ± 3.14
	Confusion–bewilderment	2.75 ± 1.45	3.48 ± 1.84	3.52 ± 1.82	3.46 ± 1.56
	Depression–dejection	0.65 ± 1.59	1.08 ± 2.36	0.58 ± 1.61	0.62 ± 1.79
	Fatigue–inertia	3.10 ± 4.55	4.38 ± 4.67	4.15 ± 4.83	4.08 ± 4.66
	Tension–anxiety	1.48 ± 2.10	1.23 ± 2.10	0.83 ± 2.12	0.92 ± 2.22
	Vigor–activity	7.98 ± 5.50	3.73 ± 4.03	3.54 ± 3.67	3.52 ± 3.92
	Total mood score	0.50 ± 11.44	7.44 ± 12.68	6.48 ± 11.43	6.73 ±11.65

**Table 6:** Means ± SDs for scores on the digit span test at four time points.

	Just before exposure	30 min into exposure	1 hr (end of exposure)	2 hr after end of exposure
Experimental	6.92 ± 1.30	6.90 ± 1.34	7.33 ± 1.40	7.46 ± 1.20
Control	6.92 ± 1.40	7.08 ± 1.25	7.46 ± 1.11	7.31 ± 1.36

**Table 7:** Number of persons self-reporting symptoms.

	Experimental			Control		
Symptom	Just before exposure	1 hr (end of exposure)	2 hr after end of exposure	Just before exposure	1 hr (end of exposure)	2 hr after end of exposure
Total headaches combined (migraine, sinus, other)	4	23	15	5	10	6
Eyes dry, irritated	2	11	7	2	3	2
Nausea	0	12	1	0	2	2
Throat sore, irritated	2	9	3	3	6	5
Throat itching inside	0	6	3	0	2	2
Tearing eyes	1	1	1	0	1	2
Sinus/nasal congestion	6	5	5	7	6	6
Nasal secretions	3	1	3	4	4	3
Nasal irritation, burning	0	1	2	0	3	1
Difficulty breathing	0	1	2	0	0	1
Cough	4	6	2	4	5	3
